# Protocol to establish a genetically engineered mouse model of IDH1-mutant astrocytoma

**DOI:** 10.1016/j.xpro.2023.102281

**Published:** 2023-05-06

**Authors:** Diana D. Shi, Joyce H. Lee, Adam C. Wang, Januka Khanal, Wenhua Gao, William G. Kaelin, Samuel K. McBrayer

**Affiliations:** 1 Department of Radiation Oncology, Dana-Farber/Brigham and Women’s Cancer Center, Harvard Medical School, Boston, MA 02215, USA; 2 Children’s Medical Center Research Institute, University of Texas Southwestern Medical Center, Dallas, TX 75390, USA; 3 Department of Medical Oncology, Dana-Farber Cancer Institute and Brigham and Women’s Hospital, Harvard Medical School, Boston, MA 02215, USA; 4 Howard Hughes Medical Institute, Chevy Chase, MD 20815, USA; 5 Department of Pediatrics, University of Texas Southwestern Medical Center, Dallas, TX 75390, USA; 6 Harold C. Simmons Comprehensive Cancer Center, University of Texas Southwestern Medical Center, Dallas, TX 75235, USA

**Keywords:** Cancer, Model Organisms, Neuroscience

## Abstract

Lower-grade gliomas exhibit a high prevalence of isocitrate dehydrogenase 1 (*IDH1*) mutations, but faithful models for studying these tumors are lacking. Here, we present a protocol to establish a genetically engineered mouse (GEM) model of grade 3 astrocytoma driven by the *Idh1*^*R132H*^ oncogene. We describe steps for breeding compound transgenic mice and intracranially delivering adeno-associated virus particles, followed by post-surgical surveillance via magnetic resonance imaging. This protocol enables the generation and use of a GEM to study lower-grade IDH-mutant gliomas.

For complete details on the use and execution of this protocol, please refer to Shi et al. (2022).^1^

## Highlights

• Use CRISPR/Cas9, adeno-associated virus (AAV), and transgenic mice to mutate neural cells
• Generate spontaneously forming murine grade 3 astrocytomas driven by mutant *Idh1*
• Apply magnetic resonance imaging (MRI) of mouse brains to study intracranial tumor growth
• Create neurosphere and allograft models of IDH1 mutant astrocytoma

## Abstract (editor highlights)

Publisher’s note: Undertaking any experimental protocol requires adherence to local institutional guidelines for laboratory safety and ethics.

## Graphical abstract

**Figure:**
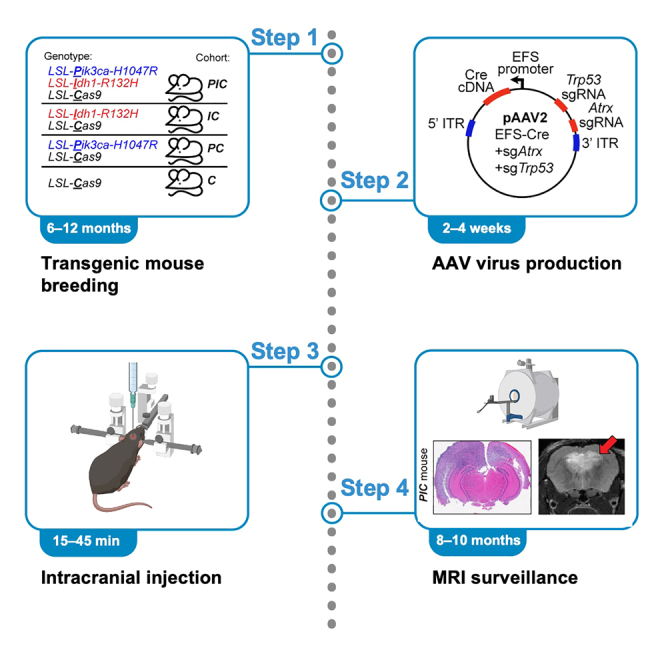

## Before you begin

This protocol describes the specific steps required to generate a genetically engineered mouse (GEM) model of grade 3 IDH-mutant astrocytoma. The tumors arising in this mouse model recapitulate key histologic and lineage features of grade 3 IDH-mutant human astrocytomas and are driven by mutant IDH. Our approach entails two primary activities: 1) breeding compound transgenic mice, and 2) delivering AAV particles into the brains of compound transgenic mice via stereotactic intracranial injections. In addition to describing how to establish this IDH-mutant glioma GEM model, this protocol also covers establishment of isogenic control GEM models in which glioma formation is infrequently observed. This protocol can also be used to produce glioma stem-like cell (GSC) lines and allograft models from autochthonous tumors that form in the IDH-mutant glioma GEM model. All of these approaches are described in recently published work.^1^

The IDH-mutant glioma GEM model described in this protocol features oncogenic ***P****ik3ca*^*H1047R*^ and ***I****dh1*^*R132H*^ transgenes and a ***C****as9* transgene. For simplicity, we refer to the triple transgenic mice as “***PIC”*** mice. In addition to ***PIC*** mice, this protocol can also be used to generate mice with ***I****dh1*^*R132H*^ and ***C****as9* alleles (***IC***), ***P****ik3ca*^*H10474*^ and ***C****as9* alleles (***PC***), and a ***C****as9* allele alone (***C***). Naïve mice are phenotypically wild-type, as all three genes are constitutively repressed by lox-STOP-lox (LSL) elements. Intracranial injection of AAV particles harboring a Cre recombinase cDNA and sgRNAs targeting *Trp53* and *Atrx* genes activates expression of *Pik3ca*^*H1047R*^*, Idh1*^*R132H*^*,* and *Cas9* genes in transduced neural cells*.* Additionally, endogenous Cas9 expression cooperates with ectopic sgRNA expression to mutate *Trp53* and *Atrx* genes in each of the transgenic mouse lines (***PIC, IC, PC,*** and ***C***). Full details describing the characterization of these models can be found in recent work published from our group.^1^ We note several important points prior to describing this protocol:

***Note:*** AAV particles are produced from the plasmid pAAV2-sgTrp53-sgAtrx-EFS-Cre (Addgene 189977)^1^ and prepared using commercial AAV packaging services (serotype: AAV-DJ) provided by Charles River (previously Vigene Biosciences).

***Note:*** Three strains of transgenic mice are used for breeding: H11^LSL-Cas9^ mice (B6;129-*Igs2*^*tm1(CAG-cas9∗)Mmw*^/J, Jackson stock number 026816), R26-Pik3ca^H1047R^ mice (FVB.129S6-*Gt(ROSA)26Sor*^*tm1(Pik3ca∗H1047R)Egan*^/J, Jackson stock number 016977), and Idh1^tm1Mak^ mice (provided by T. Mak at University of Toronto^2^^,^^3^). These mice are interbred to generate compound transgenic mouse strains used for intracranial injections in this protocol.

***Note:*** Special equipment is required that should be obtained before initiating this protocol. Specifically, this protocol requires the use of a stereotactic frame for intracranial injections (see key resources table). In addition, a small hand-held drill is required. We use a Dremel 1100 drill with a 105 engraving tip (1/8″ shank), though other drills of similar caliber may be used.

***Note:*** All surgical tools should be sterilized prior to use.

### Institutional permissions

All care and treatment of experimental animals are carried out in strict accordance with Good Animal Practice as defined by the US Office of Laboratory Animal Welfare and approved by the Dana-Farber Cancer Institute (protocol 04–019) or the UT Southwestern Medical Center (protocol 2019–102795) Institutional Animal Care and Use Committee. Any users of this protocol should ensure that the procedures outlined are performed with the corresponding permissions of the local institution.

### Generation of compound transgenic mouse strains

**Timing: 6–12 months**

**Figure 1:**
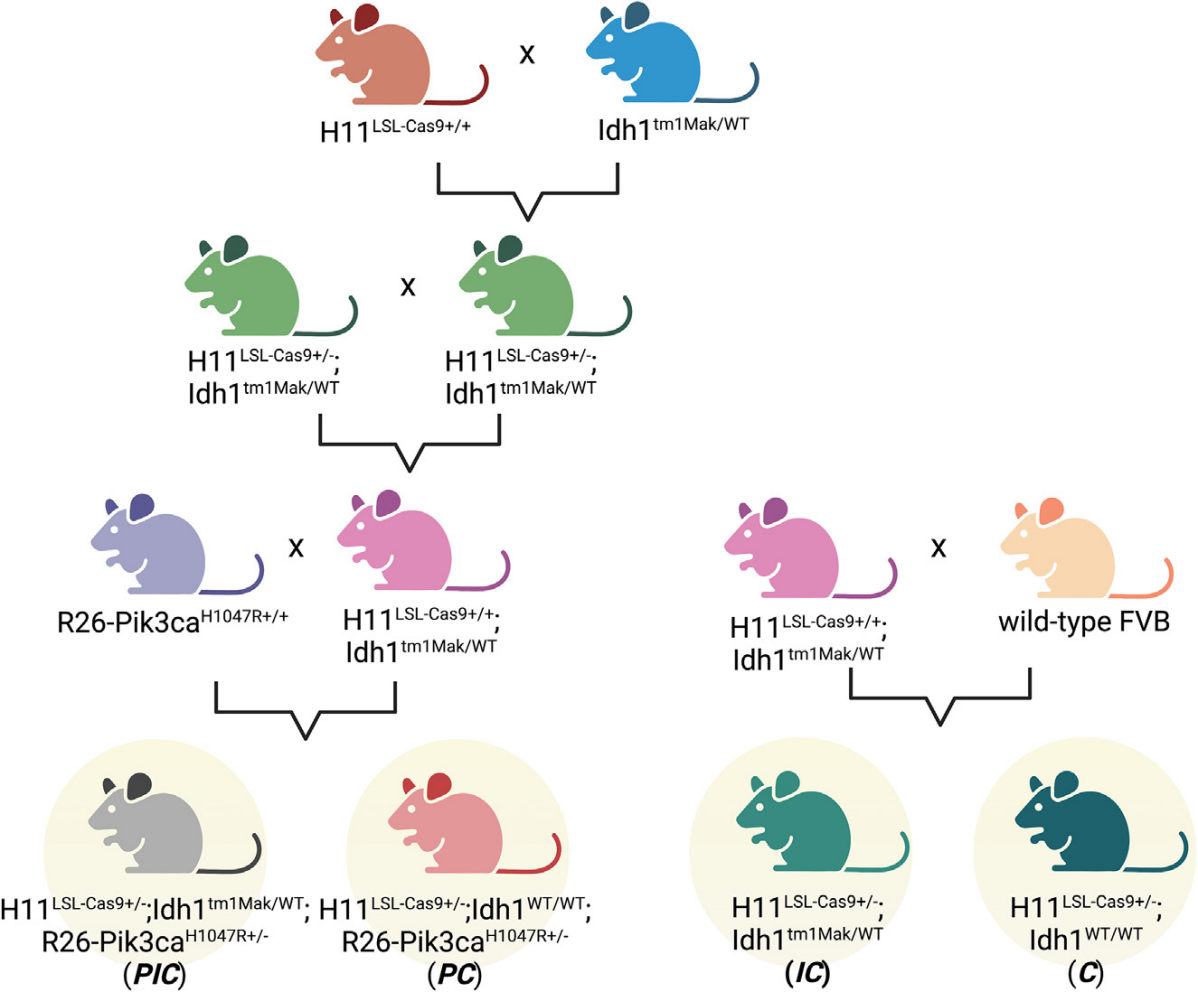
Breeding scheme Breedings required to generate ***PIC*** mice for intracranial injection of AAV. Note that this breeding scheme also generates ***PC*** mice, which can be used to generate an isogenic IDH1-wild-type control if desired. Breedings to generate ***IC*** and ***C*** mice (bottom right) are optional though can be used for additional controls. Also note that some of the depicted matings may generate multiple genotypes not shown, and the progeny of interest should be determined through the genotyping steps outlined in “generation of compound transgenic mouse strains.”

The following breeding steps are outlined in Figure 1.

1. To generate mouse strains harboring the three relevant transgenes (LSL-Cas9, LSL-Pik3ca^H1047R^, and LSL-Idh1^R132H^), first obtain H11^LSL-Cas9^ mice, R26-Pik3ca^H1047R^ mice, and Idh1^tm1Mak^ mice.
2. Breed H11^LSL-Cas9+/+^ mice (homozygous for the LSL-Cas9 knock-in allele at the H11 locus) with Idh1^tm1Mak/WT^ (heterozygous for the IDH1^R132H^ mutation at the endogenous locus) mice to produce H11^LSL-Cas9+/−^;Idh1^tm1Mak/WT^ progeny.
  a. Genotype the H11 locus using the Cas9-5 Tg and Hipp11-1 WT Transnetyx probes.
  b. Genotype the Idh1 locus using the IDH1-5 MUT Transnetyx probes (detects both IDH1^WT^ and IDH1^R132H^ and distinguishes between the two).
3. Interbreed H11^LSL-Cas9+/−^;Idh1^tm1Mak/WT^ progeny generated in step 2 to produce H11^LSL-Cas9+/+^;Idh1^tm1Mak/WT^ progeny. Verify mutational status via genotyping as described in step 2.
4. Breed H11^LSL-Cas9+/+^;Idh1^tm1Mak/WT^ mice generated in step 3 with R26-Pik3ca^H1047R+/+^ (homozygous for the LSL-Pik3ca^H1047R^ allele at the R26 locus) to produce ***PIC*** (H11^LSL-Cas9+/−^;Idh1^tm1Mak/WT^;R26-Pik3ca^H1047R+/−^) mice.
  a. This breeding will also generate ***PC*** (H11^LSL-Cas9+/−^;Idh1^WT/WT^;R26-Pik3ca^H1047R+/−^) mice, which can be used in subsequent steps to generate an isogenic IDH1-wild-type control GEM model if desired.
  b. To make additional isogenic control GEM models lacking the Pik3ca^H1047R^ transgene, cross H11^LSL-Cas9+/+^;Idh1^tm1Mak/WT^ mice with wild-type FVB mice. This breeding produces ***IC*** (H11^LSL-Cas9+/−^;Idh1^tm1Mak/WT^) and ***C*** (H11^LSL-Cas9+/−^;Idh1^WT/WT^) mouse strains. Verify genotype with progeny as above in step 2.
  c. Genotype the R26 locus using the Pik3ca-1 Tg and ROSA WT Transnetyx probes.

***Note:*** Stereotactic intracranial AAV injections, described later in this protocol, should be performed on male and female mice at least 6 weeks and no more than 7 months old. Use of younger mice may negatively affect reproducibility of injections and subsequent tumor formation due to changes in brain architecture during development.

### Preparation of AAV

**Timing: 2–4 weeks**

5. Propagate pAAV plasmid pAAV2-sgTrp53-sgAtrx-EFS-Cre that can be used to produce AAV.
6. High-titer AAV preparations can be produced through commercial sources or in individual labs. We have used Charles River (previously Vigene Biosciences) for AAV production. Viral titers should be in the range of 1 × 10^13^–1 × 10^14^ genome copies/mL.

**CRITICAL:** The titer of AAV preparations should be in the 1 × 10^13^–1 × 10^14^ genome copies/mL range. The volume that can be injected intracranially is limited to approximately ≤3 μL, and high-titer virus is thus needed to ensure adequate infection rates of neural stem and progenitor cells in the subventricular zone, the presumed cell of origin for autochthonous gliomas in this GEM model.

### Preparation of materials used for surgery

**Timing: 1 h**

7. Sterilize all surgical tools.
  a. Place surgical scissors, drill bits, syringe/needles, forceps, stapler, and staples in autoclavable container.
  b. Use a gravity autoclave setting at 121°C for a minimum of 30 min.
  c. Remove tools and let cool to room temperature (20°C–23°C).
8. Prepare aliquots of sterile PBS.
  a. Prepare sterile 1.5 mL Eppendorf tubes with 1 mL aliquots of sterile PBS inside a laminar flow hood. These will be used for flushing the syringe/needle used for intracranial injection between injections. Prepare at least one aliquoted tube per mouse to be injected.
  b. Separately, prepare a 50 mL tube of sterile PBS inside a laminar flow hood. This will be used for rinsing surgical tools (scissors, forceps) between mice.
9. Prepare aliquots of iodopovidone-based disinfectant.
  a. Prepare 1 mL aliquots of iodopovidone disinfectant solution (such as Wescodyne®) in sterile Eppendorf tubes. These will also be used to clean the syringe/needle used for intracranial injection between injections. Prepare at least one aliquoted tube per mouse to be injected.
  b. Separately, prepare a 50 mL tube of iodopovidone disinfectant solution. This will be used for disinfecting surgical tools (scissors, forceps) between mice.
10. Aliquot AAV (1 μL per mouse) into a sterile Eppendorf tube. Store AAV on ice throughout surgeries. AAV should be stored as single-use aliquots at −80°C long-term and should not undergo multiple freeze-thaw cycles.

**Pause point:** Protocol can be paused here for days to weeks before day of surgery.

### Setup of anesthesia, surgery, and recovery areas

**Timing: 30 min**

For the subsequent steps, use appropriate personal protective equipment, including sterile surgical gloves, surgical mask, surgical cap or hair net, and lab coat or disposable gown.

**Figure 2:**
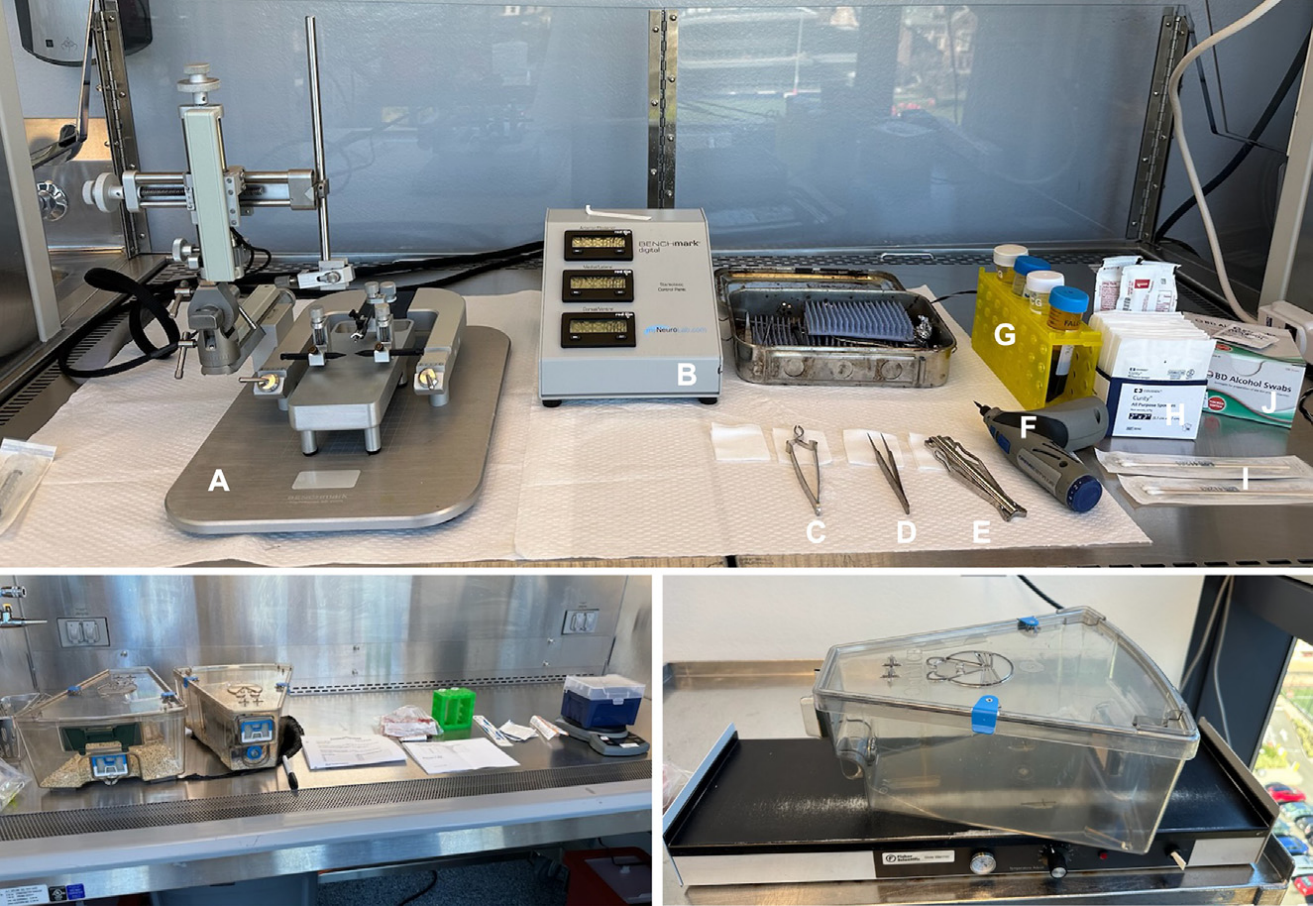
Surgical setup Top: Surgical hood setup. (A) stereotactic frame; (B) coordinate console (attached to stereotactic frame); (C) staple remover; (D) forceps; (E) stapler; (F) hand-held drill; (G) 50 mL conical tubes with iodopovidone disinfectant and empty tubes for waste collection; (H) sterile 2 × 2 gauze pads; (I) sterile cotton-tipped applicators; (J) alcohol pads. Bottom left: Hood setup for administration of anesthesia/analgesia. Bottom right: Recovery cage set on slide warmer set to 35°C–38**°**C.
12. Prepare separate, adjacent hood for anesthesia/analgesia administration (Figure 2, bottom left).
  a. Use appropriate personal protective equipment.
  b. Sanitize the surfaces of the hood with a suitable disinfectant.
  c. Prepare an empty cage to be used for holding mice immediately prior to ketamine/xylazine anesthesia administration.
  d. Prepare sterile cotton-tipped applicators and ophthalmic ointment.
  e. Set up a scale and box that can be used to weigh mice.
13. Prepare cages for surgery/anesthesia recovery (Figure 2, bottom right).
  a. Set up a slide warmer set to 35°C–38°C with 1–2 empty cages to be used for immediate post-surgical recovery.
  b. Set up a clean cage with food and antibiotic-containing water (ex. enrofloxacin). Post-surgical mice should be given antibiotic-containing drinking water for 7 days postoperatively for infection prophylaxis.

***Note:*** The above setup designates one hood for surgery preparation and anesthesia administration and a second, adjacent hood for intracranial injections. Alternative hood setups such as a single hood may be considered, for example if using inhaled anesthetics (e.g., isoflurane) to anesthetize mice.

11. Prepare the laminar flow hood to perform intracranial injections (Figure 2, top).
  a. Sanitize the surfaces of the hood with a suitable disinfectant.
  b. Plug in and power on the stereotactic frame and the control panel.
  c. Remove the surgical tools (forceps, scissors, stapler, and staples) from the box used to autoclave. Lay tools out on sterile gauze in the hood.
  d. Assemble a guide needle (3 mL syringe with a 25 G needle attached) and lay it on sterile gauze in the hood.

## Key resources table

**Table:** 

REAGENT or RESOURCE	SOURCE	IDENTIFIER
**Bacterial and virus strains**		

sgTrp53-sgAtrx-EFS-Cre AAV virus	Packaged and produced by Charles River Laboratories	N/A

**Recombinant DNA**		

pAAV2-sgTrp53-sgAtrx-EFS-Cre	Addgene^1^	Ca# 189977

**Experimental models: Organisms/strains**		

H11^LSL-Cas9^ mice (B6;129-*Igs2*^*tm1(CAG-cas9∗)Mmw*^/J), >6 weeks old males and females	Jackson Laboratories	Cat# 0268166
Pik3ca^H1047R^ mice (FVB.129S6-*Gt(ROSA)26Sor*^*tm1(Pik3ca∗H1047R)Egan*^/J), >6 weeks old males and females	Jackson Laboratories	Cat# 016977
Idh1^tm1Mak^ mice, >6 weeks old males and females	Provided by T. Mak, University of Toronto	N/A
ICR SCID mice, 6–12-week old females	Taconic	Cat# ICRSC-F

**Software and algorithms**		

Bruker Paravision 6.0.1 software	Bruker	https://www.news-medical.net/ParaVision-6-Preclinical-MRI-Software-from-Bruker

**Other**		

Fine scissors – sharp	Fine Science Tools, Inc.	Cat#14060-09
Straight-blade operating scissors	Thermo Fisher	Cat# 13-806-2
Dissecting Jewelers Microforceps (Curved)	Thermo Fisher	Cat# 08-953F
Dissecting Jewelers Microforceps (Straight)	Thermo Fisher	Cat# 08-953E
Autoclip™ Wound clip applier	Thermo Fisher	Cat# 01-804
Autoclip™ Wound Closing Staples	Thermo Fisher	Cat# 01-804-5
Sterile syringe, single use, 1 mL	Becton, Dickinson and Company	Cat# 309659
Sterile syringe, single use, 3 mL	Becton, Dickinson and Company	Cat# 309657
Needle, 1/2 in, 27 G	Becton, Dickinson and Company	Cat# 305109
Needle, 5/8 in, 25 G	Becton, Dickinson and Company	Cat# 305901
Insulin syringes, 3/10 mL, 12.7 mm, 29 G	Becton, Dickinson and Company	Cat# 324702
Needle, 1.5 in, 32 G	Hamilton Company	Cat# 7803-04
5 μL syringe	Hamilton Company	Cat# 87930
Cleaning concentrate	Hamilton Company	Cat# 18310
Tungsten needle cleaning wires	Avantor	Cat# 72310-064
28 mm diameter syringe filters, 0.2 μm Pore	Corning	Cat# 431219
Stylus Lithium-Ion (1100-25) Drill	Dremel	Cat# F0131100JA
Dremel 105 Engraving bit	Dremel	Cat# 26150105AE
Gauze sponges, 2 × 2	Covidien	Cat# 8042
Gauze sponges, 4 × 4	Thermo Fisher	Cat# 22-415-469
50 mL conical centrifuge tubes	Thermo Fisher	Cat# 14-959-49A
1.5 mL microcentrifuge tubes	USA Scientific, Inc.	Cat# 1615-5500
Wescodyne® One-Step Germicidal Detergent (iodopovidone disinfectant)	Steris Corporation	Cat# NJ-138
Cotton tipped wood applicators	MediChoice	Cat# 1314WOD1004
Sterile DPBS, no calcium, no magnesium	Gibco	Cat# 14190144
Sterile alcohol pads	Medline	Cat# MDS090670
Ketamine/xylazine cocktail, 22 mg/mL ketamine, 2.2 mg/mL xylazine	Patterson Veterinary	Cat# 07-803-6637
Ethiqa™ (buprenorphine XR)	Fidelis Animal Health	N/A
Meloxicam, 0.5 mg/mL meloxicam	Pivetal	Cat# 21294589
Ophthalmic ointment	Dechra Veterinary Products	N/A
Digital Stereotaxic Instrument	Leica Biosystems	Cat# 39477001
Slide wwarmer	Thermo Fisher	Cat# 12-594
Neural Tissue Dissociation Kit	Miltenyi	Cat# 130-092-628
Bruker BioSpec 7T/30 cm USR horizontal bore Superconducting Magnet System	Bruker	https://www.bruker.com/en/products-and-solutions/preclinical-imaging/mri/biospec/biospec-70-30-and-94-30.html

## Materials and equipment

**Table:** Ketamine xylazine anesthetic solution

Reagent	Final concentration	Amount
Ketamine (100 mg/mL)	28 mg/mL	280 μL
Xylazine (100 mg/mL)	2.4 mg/mL	24 μL
Sterile PBS	N/A	696 μL
**Total**	**N/A**	**1000 μL**

Store at room temperature (20°C–23°C) for up to one week.

## Step-by-step method details

The protocols below detail the component steps for stereotactic intracranial injections of AAV. Variations in the steps are noted when injecting cells to generate GEM-derived allografts (see generation of GEM-derived allografts).

### Anesthetizing the mouse

**Timing: 10–30 min**

The following steps detail the procedures for administering injectable ketamine/xylazine anesthetic and for assessing depth of anesthesia.

**Figure 3:**
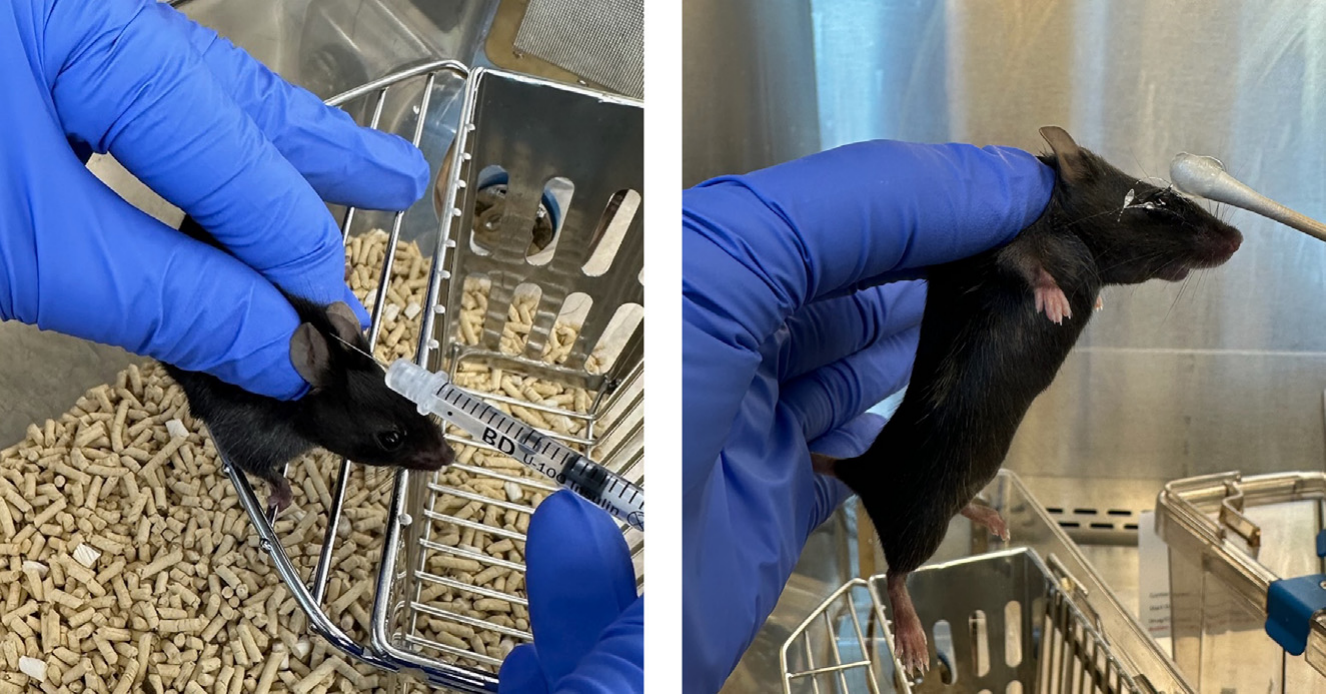
Application of local anesthetic and ophthalmic ointment Left: Administration of meloxicam local anesthetic. Right: Administration of ophthalmic ointment.

1. Prepare the ketamine/xylazine anesthetic solution
  a. Make the ketamine/xylazine solution as described in the materials and equipment section of this protocol.
  b. Filter sterilize solution by passing the solution through a 0.22 μm filter with a syringe.
  c. Weigh mouse to be anesthetized. Record weight (in grams) and return mouse to cage.
  d. Use a 1 mL syringe to slowly draw up volume of ketamine/xylazine mixture at 5 μL per gram of body weight, achieving a final dose of 140 mg/kg ketamine and 12 mg/kg xylazine.
  e. Turn the syringe needle up and plunge the syringe to expel any air bubbles in the syringe.
  f. Set syringe down on a clean surface. Use caution with the exposed needle tip.
2. Administer the ketamine/xylazine solution
  a. Hold and stabilize the mouse in one hand with a controlled and firm grip.
  b. Use an alcohol pad to sterilize the intraperitoneal injection site.
  c. Anesthetize the mouse by injecting it with the pre-filled 1 mL syringe intraperitoneally.
  d. Place mouse in separate clean cage to allow for anesthesia to take effect.
3. Assess depth of anesthesia
  a. The mouse should exhibit slowed movements within 1–2 min of injection.
  b. Begin checking for depth of anesthesia approximately 5–10 min following injection. Perform toe pinch test and assess for presence of reflexive movements after a firm pinch to one of the hind feet of the mouse. Adequate depth of anesthesia may take 15 min or longer following injection.
4. Once mouse is fully anesthetized, apply ophthalmic ointment to both eyes (Figure 3, right) and use clippers to shave the fur above the skull. Duration of anesthesia is approximately 1–2 h.

**CRITICAL:** Ensure that the mouse is fully anesthetized and does not move in response to the toe pinch test. It is important to anesthetize the mouse fully prior to performing intracranial injections. The skull is well-innervated, and this procedure can cause pain if the mouse is not fully anesthetized. Intracranial surgeries performed without sufficient anesthesia can cause movement during skull drilling and undue pain and suffering to the mouse.

***Note:*** We utilize ketamine/xylazine, extended-release buprenorphine, and meloxicam for anesthesia/analgesia for these surgeries, although alternative anesthetics may be used (e.g., isoflurane).

5. Administer local anesthetic
  a. Using previously recorded weight of mouse, draw up appropriate volume of 0.5 mg/mL meloxicam in 1 mL syringe to achieve 0.5 mg/kg dose.
  b. Turn syringe needle up and expel any bubbles in syringe.
  c. Administer meloxicam subcutaneously to the scruff between the ears (Figure 3, left).

### Intracranial injection

**Timing: 5–15 min**

These steps include positioning of the mouse into the stereotactic frame and intracranial injection of AAV.

**Figure 4:**
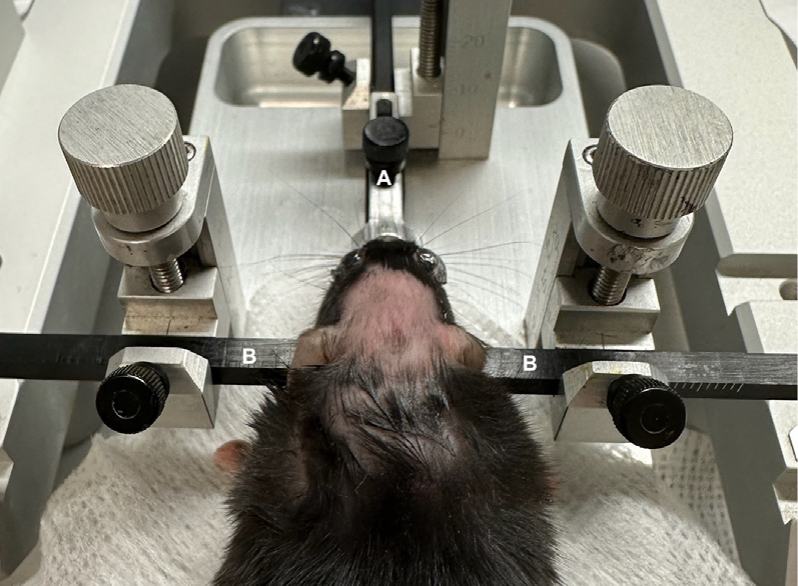
Positioning in stereotactic frame Anesthetized mouse positioned in stereotactic frame with ear pins and nose stabilizer in place. (A) Nose piece; (B) ear pins.
  c. Position ear pins into the mouse. See troubleshooting 1.
  d. The mouse’s head should be securely immobilized in the stereotactic frame. Test this by applying gentle but firm downward pressure onto the mouse’s head. The mouse’s head should remain in place when firm pressure is applied.

6. Position anesthetized mouse into stereotactic frame.
  a. Place anesthetized mouse onto the stage.
  b. Loosen screw on the nose positioning piece (Figure 4) and hook front teeth of mouse into the designated bite hook. Tighten screw on nose piece.

**CRITICAL:** Mouse positioning should be very stable prior to proceeding to next step. If the mouse is not securely positioned into the stereotactic frame, the mouse may move during skull drilling, leading to inaccurate burr hole creation.

**Figure 5:**
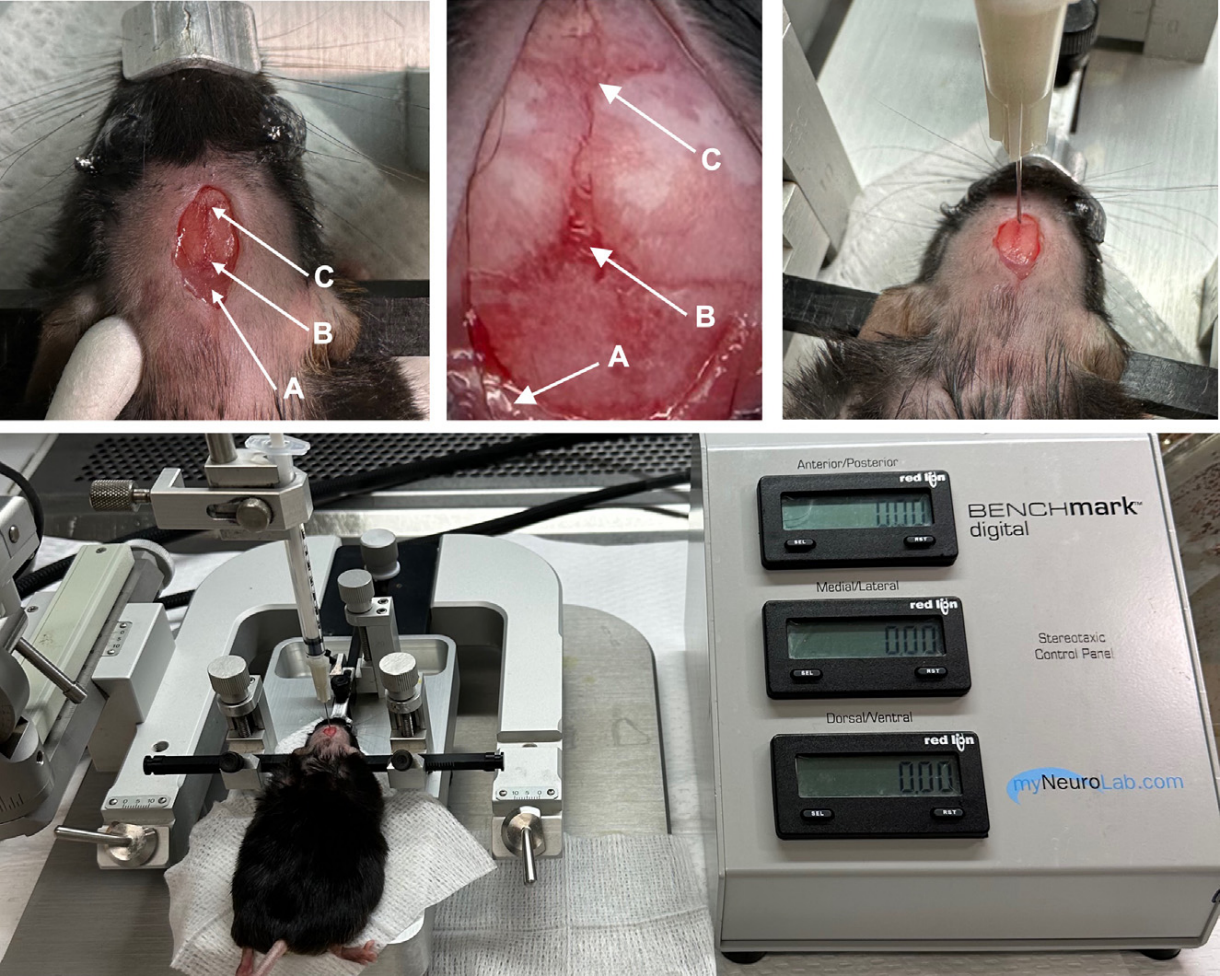
Anatomical landmarks Anesthetized mouse positioned in stereotactic frame. Guide needle is positioned on the bregma with coordinates zeroed. (A) outer membrane, removed from exposed skull surface; (B) Lambda: (C) Bregma.
9. Use a cotton-tipped applicator and gentle pressure to rub the outer membrane away from the exposed skull (Figure 5, top left).

***Note:*** The membrane is clear and will feel slick. The underlying skull will feel noticeably less slick and rougher when rubbed with the cotton-tipped applicator.

7. Use an alcohol pad to disinfect the skin surface overlaying the skull.
8. Make a 1–1.5 cm incision in the craniocaudal direction to expose the skull (Figure 5).

10. Position the guide needle (3 mL syringe with a 25 G needle attached) into the syringe holder on the stereotactic frame (Figure 5, top right).

***Note:*** The guide needle is used to zero the coordinates on the stereotactic frame. The coordinates from this positioning will be used to determine the site of virus injection.

11. Position the needle directly centered on the bregma (Figure 5, top right).

***Note:*** A detailed image of the exposed skull anatomy is included in Figure 5 (top middle) to guide visualization of the anatomical landmarks. The guide needle tip may gently touch the skull to confirm accurate positioning, but do not pierce the skull with the guide needle.

12. Zero the anterior/posterior and medial/lateral coordinates on the stereotactic frame (Figure 5, bottom).

**CRITICAL:** Ensure that the guide needle is positioned precisely at the location of the bregma. Inaccurate zeroing of the coordinates at the bregma may lead to inaccurate viral injection site.

13. With the guide needle still in place, use the stereotactic frame adjustments to move the guide needle 1 mm posterior and 1 mm lateral to the bregma.
14. Drill a burr hole into the skull at this coordinate position.
  a. Position the tip of the guide needle 0.5–1 cm above the surface of the skull to indicate injection site.
  b. Use the handheld drill to carefully drill a small burr hole at this location on the skull (1 mm poster and 1 mm lateral to the bregma) (Figure 6, left, middle). See troubleshooting 2.

**Figure 6:**
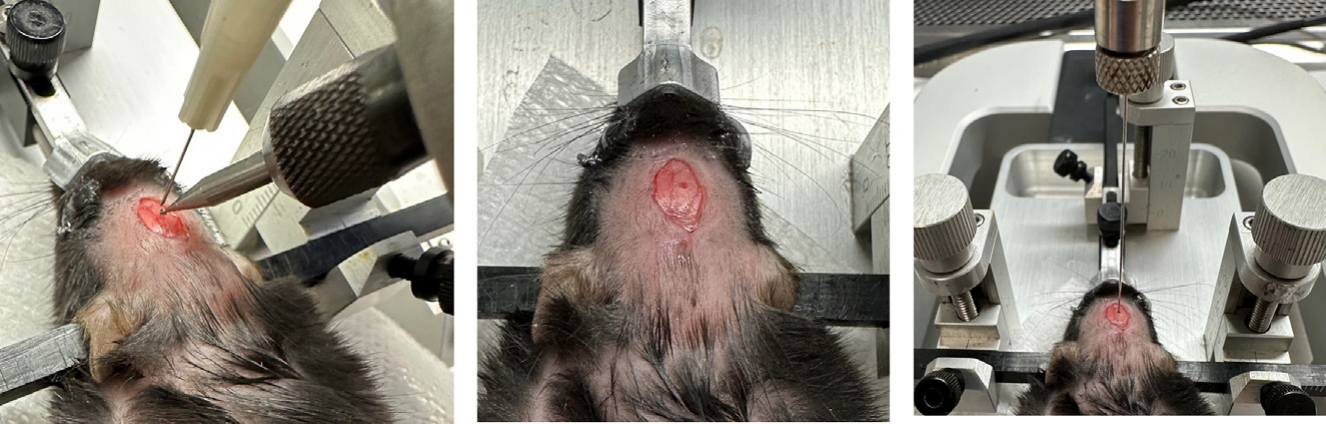
Intracranial injection Left: handheld drill with guide needle positioned 1 mm lateral and 1 mm posterior to the bregma; middle: burr hole formed following drilling into skull; right: 5 μL syringe used to intracranially inject 1 μL virus.
  c. Apply gentle pressure and retract drill bit as soon as pressure abates, indicating successful passage of the drill bit through the skull.
15. Using a 5 μL syringe with affixed 1.5 in, 32 G needle, carefully draw up 1 μL AAV virus. Carefully inspect the syringe to confirm absence of bubbles.

**CRITICAL:** Ensure that there are no bubbles present in the syringe before proceeding to next step (see troubleshooting 3).

***Note:*** Different injection coordinates are used when establishing GEM-derived allografts (3 mm anterior and 2 mm lateral to the lambda, 2.5 mm depth). See generation of GEM-derived allografts. A different volume of cells may be used to inject a total of 1 × 10^5^ cells, though this volume should not exceed 3 μL.

16. Remove the guide needle from the stereotactic frame and replace it with the 5 μL syringe/needle filled with 1 μL virus.
17. Position the syringe such that the needle is directly overlaying the hole drilled into the skull (Figure 6, right).
18. Lower the syringe/needle using the adjustments on the stereotactic frame such that the needle tip abuts the surface of the brain. Zero the dorsal/ventral coordinates on the stereotactic frame.
19. Lower the needle to a depth of 2.1 mm below the brain surface.
20. Slowly inject the virus into the brain (1 μL of virus over 3 min). Wait 1 additional minute after fully dispensing AAV particles to allow liquid to disperse in tissue before retracting needle.

**CRITICAL:** Injecting the virus slowly is important for minimizing the volume of AAV that leaks out of the brain. Excess leakage may contribute to formation of needle-track skull-based tumors.

**Figure 7:**
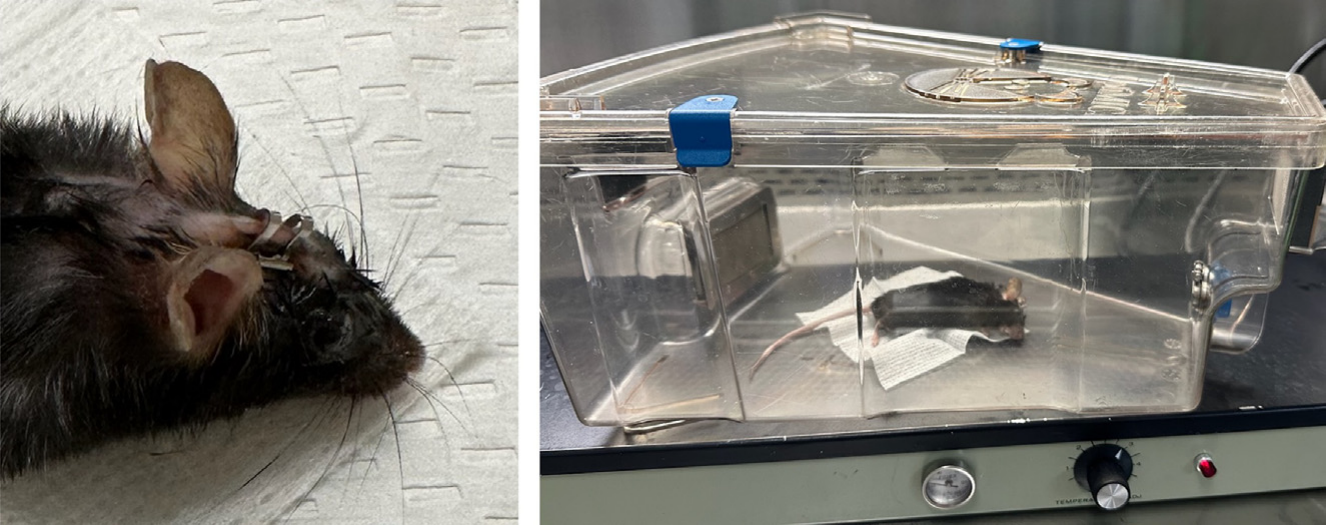
Surgical recovery Mouse with staple at injection site and in recovery cage on slide warmer.

***Note:*** Do not apply saline or other lubricants to the skull surface, as this may cause diffusion and leakage of the injected virus throughout the skull surface.

21. Slowly remove the needle from the brain by manually using the dorsal/ventral controls of the stereotactic frame over a duration of approximately 60 s. Once the needle tip is visible outside of the brain and skull, the needle and syringe can be removed from the frame.
22. Close the incision site with a wound stapler (Figure 7, left). Use forceps to bring together the skin surface in order to approximate the skin prior to applying staples.

23. Place the mouse in the recovery cage on slide warmer (Figure 7, right).
24. As the mouse begins to wake from anesthesia and spontaneously move, draw up appropriate volume of buprenorphine in a 1 mL syringe to inject the mouse with 0.1 mg/kg subcutaneously.

***Note:*** If using Ethiqa™ (extended release buprenorphine), it is recommended to use a 20 G or 23 G needle, as the suspension is viscous.

25. Sterilize buprenorphine injection site with an alcohol pad and inject buprenorphine subcutaneously.
26. Place mouse in clean cage with an antibiotic-containing water (ex. enrofloxacin). Post-surgical mice should be given antibiotic-containing drinking water for 7 days postoperatively for infection prophylaxis. Staples should be removed 7–14 days after intracranial surgery.

### Cleaning surgical instruments and inactivating leftover AAV particles

**Timing: 5 min**

These steps outline the best practices for disinfecting surgical tools between each mouse operation, as well as long-term maintenance of the 5 μL syringe to prevent clogs.

#### Surgical instruments

27. Rinse all surgical tools (scissors, forceps, drill bit) with iodopovidone disinfectant, followed by sterile PBS.
28. Use sterile gauze to dry them.

#### Intracranial injection syringe

29. Dip the needle tip of the 5 μL syringe into an aliquot of povidone-iodine disinfectant, followed by sterile PBS.
30. Dry needle tip thoroughly with sterile gauze. Use caution to not bend the needle tip.
31. Draw up and expel sterile PBS 3–5 times to flush contents of syringe.

***Note:*** If syringe does not draw up or draws up with bubbles, remove the plunger, back fill syringe with sterile PBS, and replace plunger.

***Note:*** To optimally preserve use of the 5 μL syringe, use cleaning concentrate solution (see key resources table) per package instructions after concluding intracranial injections.

#### Inactivate AAV particles

32. Expose all solutions containing AAV particles and all solutions that have come in contact with AAV particles to 10% bleach for >15 min to inactivate virus. After inactivation, solutions can be discarded via sink disposal.

### Post-surgical MRI surveillance

**Timing: 8–10 months**

This section describes steps necessary for MRI surveillance of mouse brains, which is used to assess for intracranial tumor growth.

33. Monitor AAV-injected mice for tumor formation via serial (monthly) magnetic resonance imaging (MRI). We detail below the MRI equipment and protocols used in our study, though alternative parameters and sequences may also be effective.
  a. Perform MRIs using a Bruker BioSpec 7T/30 cm USR horizontal bore Superconducting Magnet System (maximum gradient amplitude of 440 mT/m and slew rate of 3,440 T/m/s and uses a 23 mm ID birdcage volume radiofrequency (RF) coil for both RF excitation and receiving).
  b. Obtain T2 weighted images of the brain using a fast spin echo (RARE) sequences with fat suppression.
  c. Use the fofllowing parameters for image acquisition:
    i. Repetition time (TR) = 6,000 ms.
    ii. Echo time (TE) = 36 ms.
    iii. Field of view (FOV) = 19.2 × 19.2 mm^2^.
    iv. Matrix size = 192 × 192.
    v. Spatial resolution = 100 × 100 μm^2^.
    vi. Slice thickness = 0.5 mm.
    vii. Number of slices = 36.
    viii. Rare factor = 16.
    ix. Number of averages = 8.
    x. Total acquisition time 7:30 min.
      ***Note:*** Bruker Paravision 6.0.1 software is used for MRI data acquisition.
  d. Identify tumors by visually identifying tumor mass on MRI scans at the general site of injection and observing growth of this mass over serial MRI scans.

***Note:*** Presence of tumor is confirmed on pathological analysis. No specific thresholds or computational analyses are performed on MRI scans to distinguish tumor from normal tissue.

### Generation of GEM-derived allografts (optional)

**Timing: 8–10 months**

These steps outline the procedures for generating IDH-mutant GEM-derived allograft mouse models.^1^ Allograft models are generated by intracranially injecting glioma stem-like cells (GSCs) harvested from tumor-bearing ***PIC*** mice into recipient mice. GSCs derived from autochthonous tumors in ***PIC*** mice grow as neurospheres *in vitro* and form orthotopic allograft gliomas readily upon intracranial implantation. Use of allograft models circumvents the formation of needle-track osteosarcomas and protracted tumor latency associated with the GEM model.^1^ The procedures for anesthesia and intracranial injection to produce orthotopic allografts are largely identical to the above outlined steps to generate the GEM. The critical points of difference are highlighted in the above protocol steps and emphasized below.

34. ***PIC*** tumor-derived GSC line creation
  a. After brain tumor formation on MRI is observed, euthanize the mouse.
  b. Immediately harvest the brain and isolate tumor-containing tissue by gross inspection.
  c. Dissociate tumor tissue into a single cell suspension using the Miltenyi Neural Tissue Dissociation Kit (see key resources table).
  d. Culture cells in 5% CO_2_ and 5% O_2_ on ultra-low adherence plates to select for neurosphere-forming GSCs.

**CRITICAL:** GEM-derived neurospheres must be cultured at 5% O_2_. Attempts to establish murine neurosphere cell lines from this GEM model under ambient oxygen conditions were unsuccessful.

35. Intracranial injection
  a. Inject 1 × 10^5^ cells (prepared in phosphate-buffered saline) into female ICR SCID mice. Note that the preparation of cells should be at a concentration of at least 3.3 × 10^4^ cells/μL so as not to exceed 3 μL injection volume.
    **CRITICAL:** Use of injection volume ≥3 μL and/or excessively rapid injections may result in leakage of cell suspension out of the brain and affect glioma latency and promote the development of ectopic tumors (see problem 5).
  b. Inject cells at 3 mm anterior, 2 mm lateral to the lambda (Figure 5) and 2.5 mm below the brain surface.
36. Monitor tumors via MRI
  a. Monitor mice for tumor formation with serial MRI scans as in step 33.

## Expected outcomes

**Figure 8:**
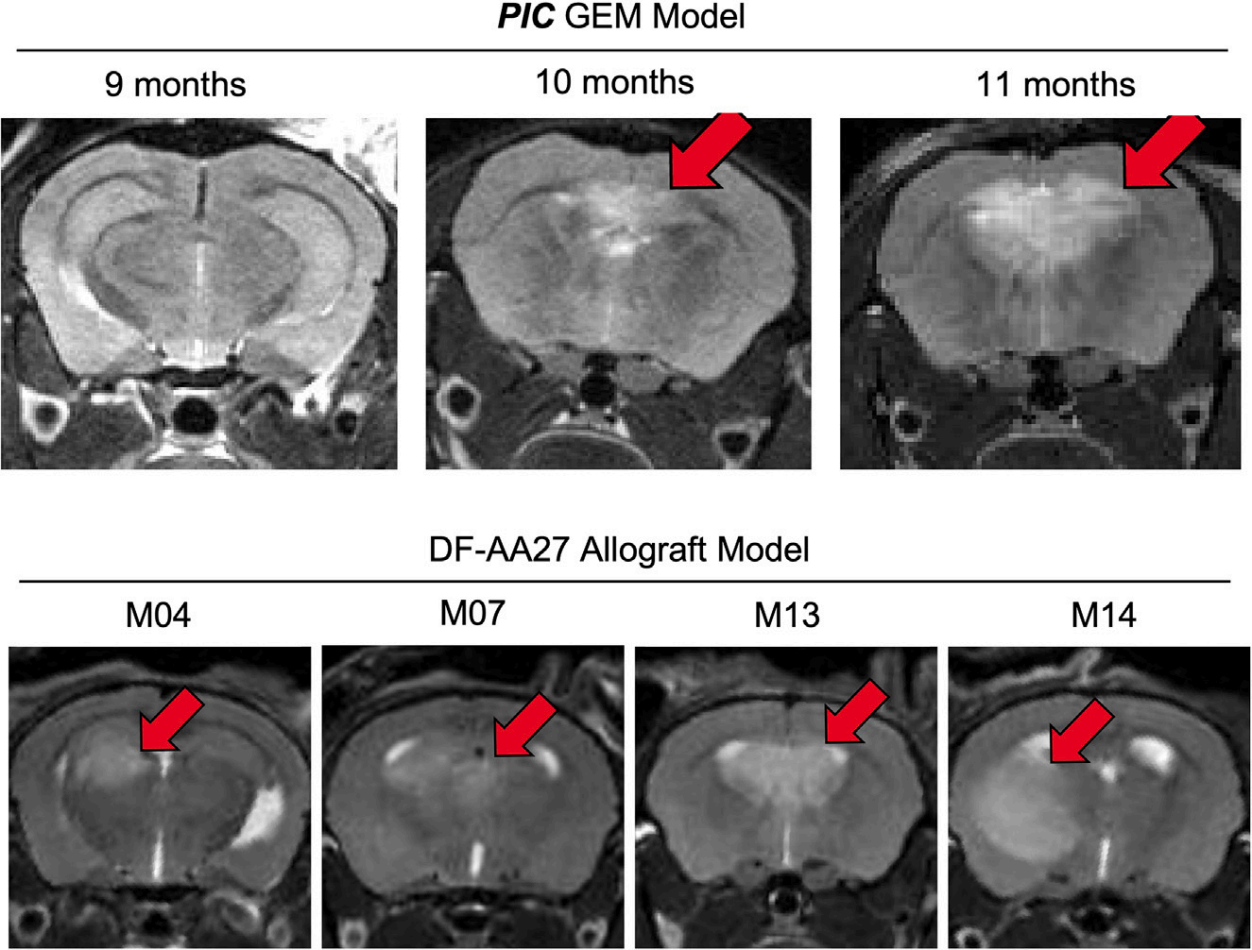
Example of magnetic resonance imaging (MRI) scan Representative MRI scan of glioma-containing mouse brains in the GEM model (top) at the indicated time points following AAV injection, and in four different DF-AA27 allograft mice (bottom). Figures originally published in Shi et al., *Cancer Cell*.^1^

Visible tumors are present on MRI scans starting as early as 8 months after AAV injection in ***PIC*** mice (Figure 8). DF-AA27 cells derived from a glioma arising in a PIC mouse^1^ formed allografts visualizable on MRI scans 1–4 months after intracranial implantation (Figure 8).

## Limitations

The latency period between AAV injection and detectable tumor formation is long (≥8 months). While this long latency period is consistent with the relatively indolent nature of lower-grade human gliomas, this time course should be considered in experimental designs using this model. Tumors are typically first detectable in their early stage on MRI scan prior to causing any neurological deficits in the mouse. However, neurological symptoms in the mouse may be an indicator of tumor growth, and thus mice should also be monitored periodically, particularly >4 months following injection. In addition, not all of the mice injected with AAV in this protocol will form gliomas. Some mice develop needle-track osteosarcoma tumors even when intracranial surgeries appeared to have been conducted optimally. To maximize accuracy of injection, we recommend performing practice surgeries on cadaver mice with trypan blue dye to confirm injection site (see troubleshooting 5). For increased tumor incidence and shorter latency, we recommend pursuing steps to generate allograft models as described above.

## Troubleshooting

### Problem 1

Difficulty in positioning the mouse onto the stereotactic frame (step 6).

### Potential solution

• If stable positioning of the mouse is difficult to achieve, it is recommended that the person performing this procedure practice on cadavers. The mouse should be first secured into the nose fastener prior to securing with ear pins. The mouse’s front teeth should fall within the bite hook (a small hole) in the nose fastener to facilitate stable positioning. The teeth should be securely inserted into the bite hook. This can be tested by gently pulling on the mouse’s tail, at which point there should be a noticeable tension preventing the mouse’s body from moving.
• If the ear pins do not stay within the mouse’s ears, it may be helpful to position them in ears and tighten both screws together (as opposed to tightening one screw fully and then the other). The ear pins should be deep enough to allow for immobilization of the mouse, but not so deep as to cause physical trauma to the mouse.

### Problem 2

Difficulty with drilling the skull (step 14).

### Potential solution

• Firm pressure should be applied with the handheld drill to pierce the skull. Pressure should be quickly released as soon as the skull has been successfully penetrated. Applying too much pressure will cause bleeding and should be avoided.

### Problem 3

Air bubbles when drawing up AAV virus (step 15).

### Potential solution

• If air bubbles are present within the syringe, carefully expel the virus back into the tube and draw up again.
• If bubbles persist, remove the plunger from the syringe and back fill the syringe with ≥5 μL sterile PBS. Visible drops of PBS should fall from the needle tip upon backfilling. If drops do not appear, it may indicate a clog in the needle or loose connection of the needle to the syringe.

### Problem 4

Lack of tumor growth.

### Potential solution

• Ensure injection site coordinates are accurate (1 mm posterior, 1 mm lateral to the bregma, 2.1 mm depth). See intracranial injection, step 13.
• Even with seeming optimal intracranial injections, some injected mice may not form tumors.

### Problem 5

Skull-based tumor forms.

### Potential solution

• Needle-track tumors may form on the skull. This may be visible as a firm mass below the skin surface on the mouse’s head that develops several months after injection.
• Ensure that depth coordinates are accurately zeroed on stereotactic frame at time of injection.
• Inject virus slowly to decrease leakage out of the brain.
• Inject low volume (∼1 μL) virus to decrease leakage out of the brain.

## Resource availability

### Lead contact

Further information and requests for resources and reagents should be directed to and will be fulfilled by the lead contact, Samuel McBrayer (samuel.mcbrayer@utsouthwestern.edu).

### Materials availability

Reagents, resources, services, and equipment are outlined in the key resources table. Unique resources used in this protocol may be requested by contacting the lead contact, Samuel McBrayer (samuel.mcbrayer@utsouthwestern.edu). pAAV2-sgTrp53-sgAtrx-EFS-Cre AAV vector is available from Addgene (Plasmid #189977).

## Data Availability

This study did not generate databases nor code.
